# Production of self-assembling biomaterials for tissue engineering

**DOI:** 10.1016/j.tibtech.2009.04.002

**Published:** 2009-07

**Authors:** Stuart Kyle, Amalia Aggeli, Eileen Ingham, Michael J. McPherson

**Affiliations:** 1 Astbury Centre for Structural Molecular Biology, University of Leeds, Leeds, LS2 9JT, UK; 2 Institute of Molecular and Cellular Biology, University of Leeds, Leeds, LS2 9JT, UK; 3 Institute of Medical and Biological Engineering, University of Leeds, Leeds, LS2 9JT, UK; 4 Centre for Self-Organising Molecular Systems, School of Chemistry, University of Leeds, Leeds, LS2 9JT, UK

## Abstract

Self-assembling peptide-based biomaterials are being developed for use as 3D tissue engineering scaffolds and for therapeutic drug-release applications. Chemical synthesis provides custom-made peptides in small quantities, but production approaches based upon transgenic organisms might be more cost-effective for large-scale peptide production. Long lead times for developing appropriate animal clones or plant lines and potential negative public opinion are obstacles to these routes. Microbes, particularly safe organisms used in the food industry, offer a more rapid route to the large-scale production of recombinant self-assembling biomaterials. In this review, recent advances and challenges in the recombinant production of collagen, elastin and *de novo* designed self-assembling peptides are discussed.

## Introduction

### The concept of self-assembly

Self-assembly is ubiquitous in nature at both macroscopic and microscopic scales and describes the spontaneous association and organization of numerous individual entities into coherent and well-defined structures without external instruction [1]. Molecular self-assembly is characterized by diffusion followed by specific association of molecules through non-covalent interactions, including hydrogen and ionic bonds, and hydrophobic and van der Waals interactions. Individually, such interactions are weak, but their large numbers will dominate the structural and conformational behaviour of the assembly [1].

In bionanotechnology, an understanding of how supramolecular architectures assemble in nature can lead to the design and synthesis of novel biomaterials. A range of complex macromolecules, macromolecular complexes and structural materials including silks [2,3], collagen [4], bones [5] and teeth [6] all display self-assembly of building blocks.

### Peptide production

Peptides and proteins have unique biological and self-assembly characteristics that are increasingly being exploited for the development of new bioactive molecules and biomaterials. There are two broad strategies for the production of peptides: chemical synthesis [7] and recombinant production by transgenic organisms ranging from bacteria and fungi to plants and animals. Chemical synthesis is rapid and effective for the production of custom-made peptides in relatively small quantities but can be costly and problematic during process scale-up and as amino acid sequence length increases; sequences over 35 amino acids are not generally considered to be economically feasible [8]. In addition, the process employs chemicals that present potential environmental hazards. Transgenic animals and plants could provide a cost-effective alternative and have been used for structural protein production. However, they are associated with long lead times, the potential for transfer of harmful animal pathogens and/or potential negative public opinion. The use of microbial ‘biofactories’ for protein synthesis is widely employed in industry owing to their ease of use, robustness and lower costs [9]. Such recombinant systems are superior to chemical synthesis routes for the production of long peptides (>35 amino acids) and proteins, although there are significant challenges for the production and efficient purification of short (10–30 amino acids) self-assembling peptides.

In this review we focus upon recombinant self-assembling protein and peptide production. Initially, we deal with natural self-assembling collagen and elastin systems, which have demonstrated utility for tissue engineering applications. Finally, we consider progress on *de novo* designed short self-assembling peptides.

## Collagen and collagen-like proteins and peptides

**Figure 1:**
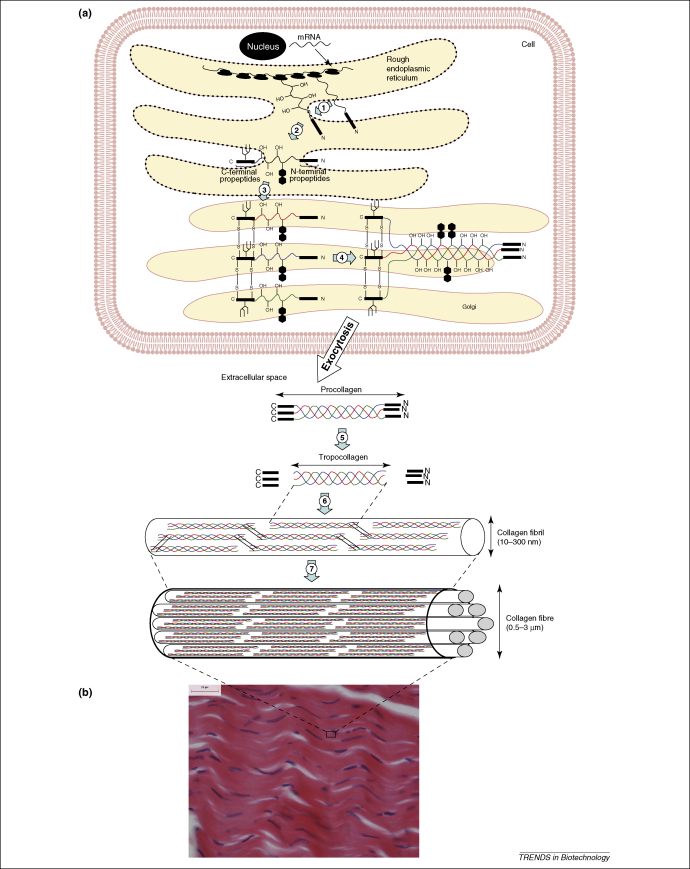
Collagen biosynthesis. **(a)** This panel shows the major events in collagen fibre assembly. Modifications of collagen include hydroxylation of prolyl and lysyl residues (1), addition of N-linked oligosaccharides (1) and glycosylation of hydroxylysyl residues in the endoplasmic reticulum (2), before chain alignment and disulphide bond formation (3), which results in the formation of the procollagen triple helix in the Golgi (4). After export from the cell, the N- and C-terminal propeptides are cleaved (5) and the resulting tropocollagen undergoes extensive crosslinking and self-assembly into collagen fibrils of diameters between 10 and 300 nm (6). These fibres in turn further assemble into larger fibres (0.5–3 μm diameter) (7). **(b)** Histological section of porcine patellar tendon showing a large number of intertwined collagen fibres with ligament fibroblasts, which are stained with haemotoxylin (stains nuclei in blue-purple) and eosin (stains collagen fibres in pink). The scale bar represents 25 μm.

The collagen superfamily is an abundant group of proteins that show high complexity, diversity, organization and function [10]. Collagens are found in all connective tissues and are a major component of the extracellular matrix. Because they constitute approximately one-third of mammalian body proteins, they are one of the most widely studied biomaterials. At least 27 different types of collagen have been identified, and all contain a characteristic triple helix tertiary structure resulting from the association of three polypeptide chains containing the sequence repeat (Gly-X-Y)_n_, where X is proline and Y is 4-hydroxyproline [11]. Collagen is synthesized and secreted from cells in the form of soluble procollagen, which is subject to modifications catalysed by procollagen metalloproteinases [12], including removal of N- and C-terminal propeptides, during the secretion and assembly process. This results in mature insoluble collagen timers (∼180 kDa). The C-terminal propeptide is essential in directing assembly of the three polypeptide chains into the triple helix structure, and its removal is a prerequisite for type I collagen fibril formation [13]. The biosynthesis of collagen and collagen fibre assembly is shown in Figure 1.

Collagen has been used for medical applications for centuries. Around 50 AD Pliny the Elder noted that ‘glue is boiled from the hides of cattle, and the best from those of bulls’ and hence collagen derives its name from the Greek words *kola*, meaning ‘glue’, and *gennan*, meaning ‘to produce’ [14]. Over the past 30 years, several collagen-based medical devices have been approved as a result of extensive safety profiling and understanding of collagen–cell interactions. Current applications include haemostasis and soft tissue repair to artificial skin, bone repair and drug delivery [15]. Collagen from animal sources has long been used in tissue engineering of heart valves, ligaments and tendons, nerves, cartilage, menisci, and blood vessels, yet the risks of pathogen transmission and immunogenicity make such sources clinically undesirable. Another challenge associated with the use of collagen as a biomaterial is its degradation, which is facilitated by extracellular matrix collagenases. Collagen can be crosslinked to decrease the rate of degradation; however, there are some concerns over poor cell infiltration, prevention of remodelling and toxicity associated with various crosslinking agents. There are obvious benefits of using recombinant production to generate safe, mechanically stable, economically viable and biocompatible collagen-like scaffolds with the potential for functionalization with bioactive groups [16,17]. Key issues in producing peptide-based collagen-like materials are that they (i) mimic the aggregating property of native collagen and (ii) include biologically active epitopes for binding integrins for collagen–cell interactions. Although higher order molecular architectures can be generated from peptide-based precursors, their physical properties are distinct from those of native collagen structures [18].

In the expression of recombinant human collagen or collagen-like polymers, the monomers should assemble to form the characteristic triple helix, which requires an expression system capable of performing appropriate post-translational modifications. To date, over 40 different genes have been identified that encode specific collagen chains, and various other genes encode determinants of collagen maturation and modification. It is thus important to identify those genes required for different physical and biological functions, such as triple helix stability, macromolecular interactions, aggregating properties, insolubility and collagen–cell interactions [18]. Several studies have demonstrated the potential of exploiting key enzymes for collagen production, including prolyl hydroxylases, lysyl hydroxylase, disulphide isomerase and lysyl oxidase. Prolyl 4-hydroxylase-catalysed modifications are crucial because they produce the 4-hydroxyproline residues that are integral to the stability of the triple helix. John *et al.* [19] were the first to use this enzyme during expression of recombinant procollagen type I in mouse milk. The mice contained cDNA constructs encoding procollagen type I and the α and β subunits of prolyl 4-hydroxylase. Levels of expression of recombinant procollagen in the milk were in the range of 50–200 mg/L [19].

An alternative to transgenic animals is expression in mammalian or insect cell lines, which should possess appropriate post-translational modification systems. There are reports of recombinant collagen expression in mammalian cells [20,21], baculoviral systems [22,23] and plant systems [24,25]. Stephan *et al.* [26] expressed recombinant α1(VIII) and α2(VIII) collagen (∼60 kDa) in mammalian cells. When secreted, these monomers formed highly stable triple-helical trimers (∼180 kDa) that further assembled into tetramers (∼700 kDa) in the presence or absence of prolyl 4-hydroxylase [26]. Limitations of such systems are that levels of collagen production are generally low and not easily detectable by western blot analysis and that cells grow relatively slowly and require complex media.

Compared with mammalian and insect cell systems, yeasts have the advantage that they can produce much larger quantities of recombinant proteins and have some of the advantages of prokaryotic hosts, such as rapid doubling times, high cell densities and growth on simple media. The methylotrophic yeast *Pichia pastoris* was engineered to express prolyl 4-hydroxylase, allowing the successful production of accurately hydroxylated triple-helical type I and III recombinant human collagen to high levels [27].

In a plant-based approach, Merle *et al.* [25] demonstrated via transient expression that hydroxylation of proline in collagen is possible by co-expression with prolyl 4-hydroxlyase α and β subunits. They then generated stably transformed tobacco plants expressing the three genes and demonstrated the production of recombinant hydroxylated homotrimeric collagen [25].

Du *et al.* [28] have produced a recombinant collagen-like protein in the bacterium *Escherichia coli*, which has advantages of rapid cell growth, high-level protein expression, simple media and low cost. The collagen-like proteins were expressed with a C-terminal foldon sequence derived from the phage T4 fibritin to stabilize the triple helix, and yields of purified proteins were around 90 mg/L culture. Cell responses to the recombinant protein were found to be better than those to native collagen, suggesting its potential for biomaterial applications [28].

## Elastin and elastin-like proteins

Elastin is an extracellular matrix protein found in connective tissue, where it provides elasticity and resilience to tissues requiring extensibility and recoil, including large blood vessels such as the aorta, lung parenchyma, ligaments, skin and elastic cartilage [29]. It is a highly insoluble, crosslinked polymer synthesized *in vivo* as soluble tropoelastin monomers (∼66 kDa), which become extensively crosslinked in the extracellular matrix, forming large complex arrays. Elastin is composed of hydrophobic domains that are rich in glycine, valine and proline residues and of crosslinking domains that are rich in lysine and alanine. The alanine residues provide spacers, allowing the display of the lysine residues [30]. The extensive covalent crosslinking of lysine residues by lysyl oxidase is important in stabilizing the polymer, which after its stabilization shows little degeneration with age [31,32].

Elastin from native sources has received less attention than collagen for biomaterial applications owing to the complexity of its purification [33] and its association with the glycoprotein fibrillin, which confers a high propensity to calcify upon implantation [34]. Hence recombinant systems for producing elastin have been established [35], and this material has been used in the form of gels or fibres as injectable scaffolds for cartilage tissue repair [36,37] and soft tissue replacement [38].

**Figure 2:**
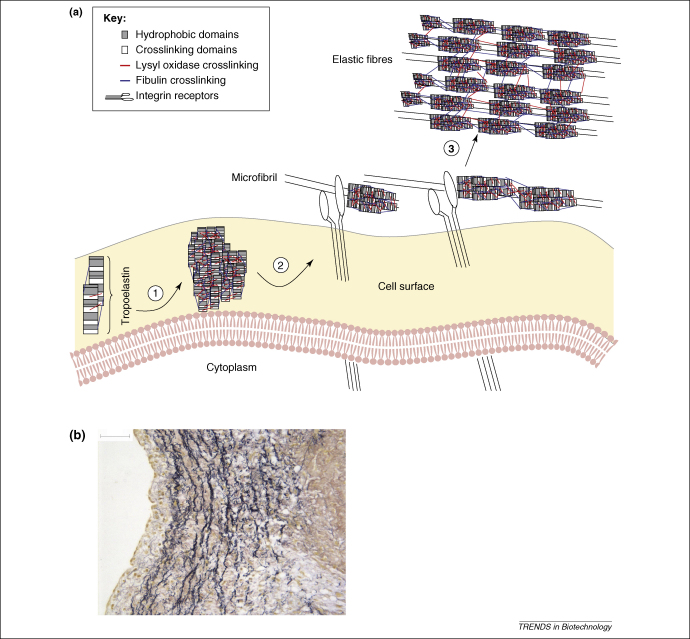
Elastin fibres. **(a)** This panel schematically illustrates the formation of elastic fibres and their assembly. After translation and prolyl hydroxylation, tropoelastin is transported to the cell surface membrane, where crosslinking by lysyl oxidase aids in tropoelastin aggregation (1). Cell surface proteins might assist the initial assembly with microfibrillar proteins, such as fibulin-4 and/or fibulin-5, thereby facilitating crosslinking. The resulting aggregates remain on the cell surface before they are transferred to extracellular microfibrils, which interact with the cell through integrins (2). Several microfibrillar proteins interact with tropoelastin, which might help in the transfer of aggregates to the microfibril. Elastin aggregates assembled on the microfibril coalesce into larger structures under possible participation of fibulin-4 and/or fibulin-5. Additional crosslinking by lysyl oxidase then leads to completion of the elastic fibre (3). Adapted from [40] with permission. **(b)** Histological section of a porcine aortic valve leaflet stained with Miller's elastin stain. The elastic fibre entanglements are shown in dark blue/black. The scale bar represents 50 μm.

The mechanism of tropoelastin monomer assembly into a polymeric matrix is not well understood, although the formation of intermediate aggregates is likely to have an organizational role in the alignment of tropoelastin monomers before crosslink formation and polymer assembly [39]. The hydrophobic domains of elastin have been shown to contribute to assembly based primarily upon the specific nature of the amino acid sequences of these domains [30]. It is evident that the self-assembly processes involved in elastin formation are complex and require further investigation. One proposed model for elastic fibre assembly is illustrated in Figure 2 [40].

Various physicochemical triggers, including temperature, pH and ionic strength, can result in tropoelastin self-aggregation by the process of coacervation [31,41]. This is a reversible process by which, for example, an increase in temperature leads to soluble protein partitioning from the solvent to an aggregated phase [42]. The lower the coacervation temperature of a molecule, then the higher will be its propensity for self-aggregation [43]. Repeats of the pentapeptide sequence VPGXG, where X is an amino acid other than proline, have been produced recombinantly as oligomeric repeats giving rise to elastin-like proteins (ELPs) with properties that make them good candidates for tissue engineering and cancer therapy applications [44]. ELPs, like natural elastin, become more ordered owing to coacervation [45].

The Urry group [39,45] has made a major contribution in the area of production of recombinant ELPs based on a VPGVG sequence with additional cell attachment sequences (GRGDSP). These ELPs proved to be biocompatible and non-cytotoxic to bovine aortic endothelial cells and to *Ligamentum nuchae* fibroblasts [39,45]. Urry and colleagues also showed that elastin-like materials containing the sequence G-(VPGVG)_19_-VPGV fused to glutathione *S*-transferase could be produced in *E. coli*. After cleavage with protease Factor Xa, the final ELP yield was 1.15 mg/L fermentation culture [46]. Betre *et al.* [36] used the temperature-induced coacervation property of ELPs at 35 °C for injectable scaffold development for cartilage repair. Below 35 °C the ELPs are soluble, thereby allowing the incorporation of cells with the ELP solution below body temperature. Upon injection at the defective site the temperature would increase and the ELPs would form a gel-like matrix encapsulating the cells. It was shown that chondrocytes maintained their rounded morphology and phenotype and produced extracellular matrix components *in vitro*. This ELP coarcervate showed comparable mechanical properties to those reported for collagen and glycosaminoglycans [36] and thus showed promise for cartilage tissue engineering. In another study, McHale *et al.* [47] designed an ELP capable of undergoing enzyme-initiated gelation catalysed by transglutaminase.

Woodhouse *et al.* [48] investigated the use of a recombinant human ELP as a coating on synthetic materials to determine whether it could improve blood compatibility of cardiovascular devices, such as vascular conduits and arterial/venous catheters. The ELP constructs were based on EP20-24-24 (∼17 kDa), which comprised exons 20, 21, 23 and 24 of human elastin and contained hydrophobic and crosslinking domains, and were produced as fusions to glutathione *S*-transferase (GST) [30] in *E. coli*.

Girotti *et al.* [49] expressed ELPs based on the sequence motif (VPGIG)_2_VPGKG, in which the lysine (K) replaced isoleucine (I) in every third repeat to allow crosslinking to occur. The ELP monomer also contained a fibronectin CS5 domain, which includes the REDV endothelial cell recognition sequence. They expressed a range of repeat sizes and purified a decamer (∼80 kDa) using an *E. coli* expression system. The purified protein was crosslinked by glutaraldehyde and resulted in insoluble hydrogel matrices [49].

Taken together, these reports demonstrate that via coacervation and further stabilization, ELPs have great potential as tissue engineering substrates. It is particularly exciting that recombinantly produced ELPs respond to physicochemical triggers and undergo transition to hydrogels. Recombinant production therefore offers a sustainable method for producing large quantities of ELPs incorporating bioactive domains and cell recognition sites.

## *De novo* designed self-assembling peptides

**Figure 3:**
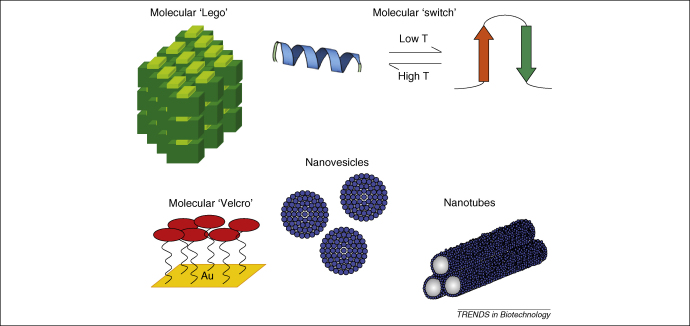
Schematic illustration of various self-assembling peptide systems [1]. Molecular ‘Lego’ forms β-sheet structures through interactions between hydrophobic and hydrophilic domains. These peptide systems are able to undergo complementary ionic bonding with the hydrophilic surface. Molecular ‘switches’ change their molecular structure upon changes in temperature. Molecular ‘Velcro’ forms monolayers on surfaces through covalent bonds between a cysteine anchor and gold atoms on the surface. Surfactant-like peptides can undergo self-assembly to form either nanovesicles or nanotubes, as shown.

During the last decade, many studies have focused on the design and use of artificial self-assembling peptides for tissue engineering [50–54] and most of these have exploited chemically synthesized peptides. Zhang and coworkers [1] have made a major contribution through their systematic investigation of peptide systems, providing insights into the chemical and structural principles that dictate the self-assembly process. The peptide systems developed include ‘molecular Lego’, which can form hydrogel scaffolds for tissue engineering, ‘molecular switches’, which can act as molecular actuators, ‘molecular hooks and Velcro’ for surface engineering, ‘molecular capsules’ for protein and gene delivery and ‘molecular cavities’ for biomineralization [1]. Schematic illustrations of self-assembling peptide systems are shown in Figure 3.

One of the first commercially available self-assembling peptides designed by Zhang was RAD16 (RADARADARADARADA), which was marketed under the name of PuraMatrix^TM^ (3DM, Inc., Cambridge, MA, USA). This 16 amino acid peptide self-assembles into a nanofibre network. It is easy to synthesize chemically and can be formulated in water at concentrations up to 50 mg/mL. Gel formation can be triggered by an increase in ionic strength or by a change in pH. This peptide has been used in a wide range of biomaterial applications, including cartilage tissue repair [55], osteoblast proliferation and differentiation [56], bone regeneration in bone defects [57] and axon regeneration [58].

Control over the process of self-assembly is crucial to the design of peptide systems that assemble and disassemble with physicochemical cues, including pH, light, ionic strength, temperature and concentration. pH switching, for example, is a relatively simple approach for controlling self-assembly. An example is the self-assembling β-peptide P_11_-4 (QQRFEWEFEQQ), which is pH sensitive due to the ionizable glutamate and arginine side chains. At concentrations below <10 mg/mL it is soluble at neutral pH but adopts a hydrogel state at low pH (pI ∼4.2) by self-assembly of anti-parallel β-sheet tapes, which then stack together to form fibrils. It will also form a hydrogel state above a critical concentration (>10 mg/mL) at pH 7.4 and a salt concentration of 140 mM in cell culture medium [59], with applications including enamel remineralization [60], injectable scaffolds [61] and joint lubricants [62]. A series of related self-assembling peptides have also been designed [63,64].

The Schneider group [65] have similarly designed MAX1 and MAX8 β-hairpin peptide systems, in which folding and hydrogel formation is initiated by the high salt content of cell culture medium at pH 7.4. In water, MAX1 is an unfolded monomer, but addition of sodium chloride to 150 mM and a pH change to 7.4 results in formation of a rigid hydrogel [65]. The P_11_ and MAX systems use an alternating hydrophobic and hydrophilic amino acid pattern to dictate a β-sheet structure via interchain hydrogen bonding.

The Woolfson group have designed peptides based on helical coiled-coils [66–68], allowing them to elucidate the design principles that underpin self-assembly of such peptide systems. They reported studies based on a heptad sequence repeat, *abcdefg*, with isoleucine and leucine at the *a* and *d* sites, respectively, ensuring coiled-coil dimerization. To direct staggered assembly of peptides and fibril formation, they incorporated lysines at the ends of the peptides with central glutamates to allow ionic interactions. The resulting fibres were straight rods, tens of microns in length, providing a good model for the development of functional biomaterials [69]. The Hartgerink group [70] have also used heptad repeats producing helical coiled-coils that form nanofibres in a concentration-dependent manner. This group also used pH and ionic strength as triggers for self-assembly with incorporation of isoleucine and leucine residues at positions *a* and *d* of the heptad, and glutamates at positions *e* and *g* providing an acidic region. Hence at low pH ionic repulsion is eliminated and carboxylic acid side chains hydrogen bond with each other [70].

The Raines and Koide groups [71,72] have developed peptide systems for creating elongating triple-helical supramolecules via intermolecular interaction between trimeric collagen-like peptides. The initial trimeric peptide assemblies were facilitated by using two [71] or three [72] peptide repeat sequences with Cys residues that were differentially positioned to allow association of a staggered arrangement of peptides into a trimer, thus providing overhanging tails for association with other trimeric units and resulting in collagen triple helices up to ∼400 nm in length that resemble natural collagen fibrils. These examples of peptides of ∼30 amino acids demonstrate a minimalist approach to developing collagen-based biomaterials with tuneable attributes by exploiting triple-helical propensity to direct self-assembly.

### Recombinant peptide production

Most of the self-assembling peptides in use have been produced through chemical synthesis, which also allows a wide range of amino acid analogues to be incorporated. Recombinant self-assembling peptide production is also being explored. The main advantages are the provision of sustainable sources of biomaterials and the ability to readily produce modified variants containing quite large bioactive motifs or domains. Biological peptide production is, however, generally restricted to using the 20 naturally occurring amino acids, although bioincorporation of unnatural amino acids into proteins and peptide is now becoming more feasible [73].

In the early 1990 s, Kuliopulos and Walsh [74] used a tandem repeat approach (Box 1) for the production of a soluble 14 aa peptide. They added peptide repeats to ketosteroid isomerase (KSI), a 125 amino acid protein that becomes localized in insoluble inclusion bodies in *E. coli*, together with a C-terminal 6His-tag for purification. The KSI-(peptide)_5_-His_6_ was expressed, purified by Ni affinity chromatography and cleaved with cyanogen bromide (CNBr) at intervening methionine residues. The peptide units all terminated with homoserine lactone derived from the C-terminal methionine. They reported a respectable yield of 50–55 mg/L of pure peptide after high performance liquid chromatography (HPLC). Although this peptide was not self-assembling, the work demonstrated the potential to produce short peptides by recombinant means. The C-terminal homoserine lactone that is formed after CNBr cleavage of the tandem repeats could, however, have adverse effects on self-assembling peptides and their bioactivity. Conversely, it could also prove beneficial in some cases by providing a mechanism for physically coupling the peptide to a solid support.

Many groups prefer not to use CNBr due to its toxicity, and instead use site-specific proteases as a cleavage system mechanism. Affinity purification with a suitable fusion tag allows recovery of the target protein/peptide, which can be released from the fusion partner by incubation with the site-specific protease. Various approaches for specific cleavage of recombinant proteins are illustrated in Table 1, and systems for protein or peptide production are shown in Table 2.

### Recombinant self-assembling peptide production

Recombinant technology has been increasingly used for self-assembling peptides. Reed *et al.* [75] used a cellulose-binding domain as a fusion partner for tandem repeats of RAD16. Glutamic acid residues were introduced before and after each repeat to allow endoproteinase GluC mediated cleavage of the peptide units. The fusion protein was affinity-purified on cellulose, a low cost matrix [75]. The main problem was the low level of recovery of purified peptide, which was only ∼5% of the expected yield. The reasons for such losses are not completely clear, but contributing factors were probably the protease cleavage step and the concentration-dependent self-assembly, which might have resulted in insoluble aggregated material.

Many natural proteins can misfold to form protein and peptide aggregates that can contribute to the pathology of diseases such as Parkinson's, Alzheimer's and type II diabetes. To understand and subsequently develop therapeutic interventions, both structural and biochemical studies of relevant peptides are necessary, demanding large quantities of the biologically relevant peptide. For example, the expression and purification of a recombinant peptide comprising residues 11–26 of the Alzheimer's β-amyloid protein (Aβ_11–26_) has been reported [76]. Three tandem repeats of Aβ_11–26_ were fused at the C-terminus of KSI, and the fusion protein was purified using Ni-affinity chromatography and HPLC, resulting in 10 mg peptide/L culture. This Aβ_11–26_ peptide formed fibrils that exhibited a similar morphology to those formed by the chemically synthesized peptide [76].

van Hell *et al.* [77] reported on the production of two self-assembling peptides designed to form vesicles in aqueous solution – SA2 (AAVVLLLWEE) and SA7 (AAVVLLLWEEEEEEE). These were produced as fusions to the SUMO protein, and the peptides were released by digestion with the site-specific SUMO protease. A 5-litre fermentation was reported to yield 300 mg of fusion protein and 30 mg of purified peptide/L culture after HPLC, which suggests 100% efficiency of peptide recovery [77].

Middelberg and colleagues [78] produced the self-assembling peptide, P_11_-2 (QQRFQWQFEQQ), which forms a concentration-dependent hydrogel. They expressed P_11_-2 as unimer, dimer, trimer or nonamer constructs fused to KSI, but rather than using CNBr cleavage of methionine, they chose cleavage of cysteine residues by 1-cyano-4-dimethylaminopyridinium tetraflouroborate. Levels of protein expression were relatively low, although recovery of 2.63 mg peptide/L culture after reverse phase (rp)HPLC nevertheless represented an efficient 42% purification of peptide [78].

**Figure 4:**
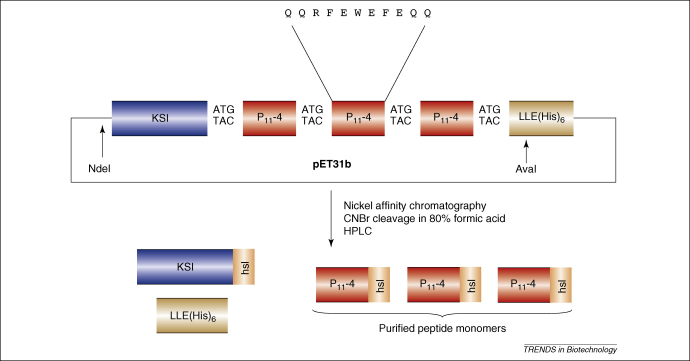
Example of the tandem-repeat strategy for high yield peptide production in the pET31b expression vector. A fusion protein comprising ketosteroid isomerase (KSI) is targeted to inclusion bodies in *E. coli* together with tandem repeat peptides encoding the P_11_-4 sequence and a His tag. The fusion protein and peptides are cleaved with cyanogen bromide (CNBr), which acts on intervening methionine residues. This results in the presence of a C-terminal homoserine lactone (hsl) on each resulting peptide monomer.

We have used KSI fusion and CNBr cleavage for recombinant production of a similar self-assembling peptide P_11_-4 as a trimer repeat [79] (Figure 4). Host strain optimization and the use of autoinduction [80] allowed enhanced levels of fusion protein and hence peptide production, resulting in the recovery of 2.5 g of fusion protein/L culture. There was loss of peptide at the purification stage because a theoretical yield of *ca.* 500 mg peptide/L culture only translated into the recovery of *ca.* 90 mg/L. Nonetheless, we believe this is the highest level of self-assembling peptide recovery so far reported from a recombinant system. This work also emphasizes that optimizing growth conditions can maximize peptide production levels. Crucially, more research is required into optimizing the approaches for efficient recovery of the expressed self-assembling peptides.

## Conclusions

The examples outlined here show that self-assembling biomaterials can act as scaffolds for tissue engineering and show tremendous promise in regenerative medicine. ‘Smart’ self-assembling peptides are able to respond to physicochemical triggers, such as concentration, pH and temperature, and are therefore ideal candidates to act as new scaffolds for tissue engineering applications. Biomaterials with the addition of cell recognition motifs, such as RGD, have already been examined in chemically synthesized peptides [81–84]. The production of short (10–30 residues) self-assembling peptides by recombinant technology is in its infancy, but these can be readily engineered to contain cell recognition sites and larger bioactive domains to provide significant benefits for tissue engineering applications. There is clear potential for the combined use of both chemically and biologically synthesized self-assembling peptides. For example, one could imagine chemically synthesized peptides providing the bulk component of a matrix supplemented by the addition of more complex self-assembling peptide-associated bioactive domains, produced by recombinant approaches, and which would integrate stochastically during the self-assembly process to provide an ideal environment for cell growth. Bioproduction of short self-assembling peptides has also been shown to be feasible using microbial systems. Even with *E. coli*, substantial amounts of peptide can be produced, although care must be taken to ensure that factors such as endotoxins are absent. Yeasts have been less well explored in this area, yet many yeast strains are capable of very high levels of protein production, and some have ‘Generally Regarded As Safe’ (GRAS) status due to their prolonged use in the food industry. In the future, however, if recombinant production can be translated into transgenic animals and particularly transgenic plants, the quantities of peptides that could be produced relatively cheaply in a sustainable manner would be essentially unlimited.

## Figures and Tables

**Table 1:** Common methods to cleave peptides at specific amino acid sequences

Cleavage agent	Cleavage specificity
**Chemical**	
Hydroxylamine	-N ↓ G-
Cyanogen bromide	-M ↓ X-
Formic acid	-D ↓ P-
2-iodosobenzoic acid	-W ↓ X-
3-bromo-3-methyl-2-(2-nitrophenylthio)-3H-indole	-W ↓ X-
2-nitro-5-thiocyanatobenzoic acid	-C ↓ X-
1-cyano-4-dimethylaminopyridiumtetrafluoroborate	-C ↓ X-
**Enzymatic**	
Enterokinase	-DDDDK ↓ X-
SUMO protease	SUMO-GG ↓ XXX-
TEV protease	-ENLYFQ ↓ (S,G)-
Factor Xa	-IDGR ↓ X-
Thrombin	-LVPR ↓ GS-
HRV 3C protease	-LEVLFQ ↓ GP-
IGase	-PP ↓ YP-
Furin	-RX(R/K)R ↓ X-
Endoproteinase Lys-C	-K ↓ X-
Endoproteinase Glu-C	-E ↓ X-
Endoproteinase Arg-C	-R ↓ X-
Endoproteinase Asp-C	-D ↓ X-
	

**Table 2:** Overview of recombinant protein/peptide production systems

Protein/peptide	Proteins/peptides expressed	Structures formed	Expression system	Yield mg/L	Refs
Homotrimeric human type I collagen	Homotrimeric collagen type I, chimeric prolyl 4-hydroxylase	Not reported	Transient expression in tobacco plants	Not reported	[24]
Procollagen	Procollagen α2 chain, and α/β subunits of prolyl 4-hydroxylase	Not reported	Transgenic mice – mouse milk	50–200	[22]
Collagen type VIII	α1(VIII) and β1(VIII) chains. Co-transfection with prolyl 4-hydroxylase	Rod-like molecules and hexagonal lattices	293-EBNA mammalian cells	Not reported	[26]
Human collagen type I and III	Substitution of a C-propeptides of proα1(I), proα2(I) and proα1(III) chains with a foldon region, co-expression with prolyl 4-hydroxylase	Not reported	*Pichia pastoris*	Not reported	[27]
Collagen-like protein	Incorporation of a T4 foldon region (GYIPEAPRDGQAYVRKDGEWVLLSTFL)	Fibrils	*Escherichia coli*	90	[38]
Elastin-like polypeptides	Derivatives of PGVGVA	Various states of coarcervation, from globular structures to fibrils	*Escherichia coli*	Not reported	[30,48]
Elastin-like polypeptides	(VGVPGVGVPGGGVPGAGVPGVGVPGVG VPGVGVPGGGVPGAGVPGGGVPG)_9_	Gel-like coarcervates	*Escherichia coli*	Not reported	[36]
Elastin-like polypeptides with incorporated cell recognition motifs	(VPGIG)_2_VPGKG(VPGIG)_2_EEIQIGHIPREDV DYHLYP(VPGIG)_2_VPGKG(VPGIG)_2_(VGVAPG)_3_	Not reported	*Escherichia coli*	Not reported	[49]
P_11_-2	QQRFQWQFEQQ	β-sheet forming fibrils	*Escherichia coli*	2.63	[78]
P_11_-4	QQRFEWEFEQQ	β-sheet forming fibrils	*Escherichia coli*	90	[79]
RAD16 peptide	RADARADARADARADAE	Nanofibres	*Ralstonia eutropha*	10.1	[75]
Aβ_11–26_ peptide	EVHHQKLVFFAEDVG	Amyloid fibrils	*Escherichia coli*	10	[76]
SA2	AAVVLLLWEE	Vesicle-forming peptides	*Escherichia coli*	6	[77]
SA7	AAVVLLLWEEEEEEE				

**Table I:** Effect of increasing peptide repeat number on theoretical yields of carrier protein and peptide

Peptide repeat number	Yield of carrier protein (%)	Yield of peptide (%)
2	84.4	15.6
3	78.3	21.7
4	73.0	27.0
5	68.4	31.6
6	64.4	35.6
9	54.6	45.4

## Box 1 Tandem repeat strategy for peptide production

The tandem repeat strategy was introduced by Shen [85] and involved peptide sequences that are separated by cleavable amino acids, usually Met or Cys. This approach was tested for proinsulin expression, which at 90 amino acids represents a small protein rather than a peptide. Unimer, dimer and trimer constructs were tested, with the trimer giving the highest yield of proinsulin. More relevant to the production of short self-assembling peptides is the strategy in which tandem repeats, again separated by a cleavable residue, are expressed as part of a fusion protein to assist in expression, stability and recovery. Because the fusion partner protein will be substantially larger than the peptide, the peptide will represent only a small proportion of the recombinant protein, and increasing the number of tandem repeats should increase the yield of peptide. For a small carrier protein of 130 amino acids fused to a single 12 amino acid peptide, the relative levels of carrier protein and peptide would be ∼91.5% and 8.5%, respectively. However, if tandem peptide repeats are included, the respective proportions of carrier and peptide are expected to alter, as shown in Table I. Unfortunately, this theoretical relationship between increasing number of repeats and their final yield is not commonly observed. Kuliopulous and Walsh [74] systematically expressed tandem repeats of a soluble 14 amino acid yeast mating factor peptide fused to a ketosteroid isomerase carrier protein. Expression levels increased up to three repeats before becoming lower up to six repeats, with a further dramatic reduction at higher numbers of repeats. For a histonin peptide, Kim *et al.* [86] tested a series of two to 32 repeats and found the highest levels of expression for a 12-mer. For self-assembling peptides, this decrease in expression of the recombinant protein with increasing number of repeats has also been observed [78,79]. For most examples of peptide production, the recombinant fusion proteins are expressed as inclusion bodies that should, in theory, protect the cell from any potential adverse effects of the peptide, as well as protecting the peptides from degradation, but perhaps the longer repeat sequences are not prevented from causing cell damage. Until the underlying reasons for the failure of expression of longer tandem peptide repeats is understood, the use of a small fusion partner and a single peptide repeat seems to be the most feasible expression strategy.
